# Cardiac Surgery to Manage Rheumatic Heart Disease in Africa Is Complex — a Geographic Perspective

**DOI:** 10.5334/gh.1394

**Published:** 2025-02-03

**Authors:** Jordan Leith, Kevin R. An, Lamia Harik, William Zhu, Michele Dell’Aquila, Taylor Brashear, Robert N. Peck, Castigliano M. Bhamidipati

**Affiliations:** 1 Department of Cardiothoracic Surgery, Weill Cornell Medicine, New York, US; 2 Center for Global Health, Weill Cornell Medical College, New York, US; 3 Department of Medicine, Bugando Medical Centre, Mwanza, Tanzania; 4 Division of Cardiothoracic Surgery, Department of Surgery, Oregon Health & Science University, Portland, Oregon, US

**Keywords:** Rheumatic heart disease, Global Cardiac Surgery, Access to Care

## Abstract

**Background:**

Rheumatic heart disease (RHD) is the most common form of acquired heart disease in Africa, often requiring surgical intervention. Previous studies have demonstrated the need for more cardiac surgeons in Africa but have not outlined their distribution relative to populations and incident cases.

**Objective:**

We estimate rheumatic heart disease incidence and cardiac surgical capacity to manage RHD in Africa. By characterizing geographic catchment areas served by the nearest cardiac surgeon(s), we estimate patient travel distance and the unmet surgical need.

**Methods:**

Subnational population, RHD incidence, cardiac surgeon, and geographic data were collected from credible, publicly accessible sources. Quantum Geographic Information System (QGIS 3.32) was used to create a subnational map of Africa and conduct nearest neighbor analyses to determine the location and distance of each subnational region’s nearest cardiac surgeon. Catchment areas were defined and characterized. Incident RHD case-to-capacity ratios (ICCR) and surgical need were calculated. Incident RHD and surgical need were mapped at the subnational level across Africa. The human development index (HDI) of each region was then incorporated to assess the impact of socioeconomic disparities.

**Results:**

A total of 779 subnational regions from 54 countries were included in the analysis. The African continent has an estimated 509 cardiac surgeons practicing in 74 subnational regions (corresponding to 74 catchment areas) and 1,027,974 incident cases of RHD annually. The average distance to travel for care by a cardiac surgeon was found to be 312.01 km (193.87 miles). The ICCR due to RHD for the African continent was 10.64.

**Conclusions:**

Access to cardiac surgical care is limited across Africa despite the high incidence of RHD. While nearly all areas of the continent would benefit from increasing cardiac surgical capacity, attention should be paid towards strategic development of geographically accessible cardiac surgical centers to equitize care for RHD.

## Introduction

Rheumatic Heart Disease (RHD) is the most common form of acquired heart disease in Africa (1), with incidences estimated at 60 times greater (2,3) than those in high-income nations like the U.S. and those in Western Europe (4,5). This difference is predominantly driven by socioeconomic living conditions and easier access to screening, antibiotics, and treatment (1). Given the structurally destructive nature of RHD to myocardial substrate, surgical intervention is the only cure (6). However, because of limited access to centers with cardiac surgery capability, 20% of Africans with RHD die by the age of 15, and 70% die by the age of 25 (1).

Compared to high-income countries, access to cardiac surgical care in low- and middle-income countries (LMICs are defined as those with a Gross National Income (GNI) per capita, calculated using the World Bank Atlas method, between $1,086 and $4,255) is limited, with countries in Sub-Saharan Africa having the fewest surgeons and hospitals (7,8). Contemporary studies assessing global access to cardiac surgical care have represented the need gap at a very macro level (7,8). In stark contrast to the U.S. population, no work delving into the incidence of RHD, characterizing geographic catchment areas, and considering travel distance for cardiac surgical care has been done for African patients (9,10). This is especially important in the African context given its geography and the potential need to optimally locate centers with cardiac surgical capabilities (U.S. land mass ~3.8 million square miles vs. Africa land mass ~11.7 million square miles).

The aim of this work was to characterize geographic catchment areas, cardiac surgeon workloads, estimate patient travel distance, and co-register the incidence of RHD to establish the unmet cardiac surgical need. Further, we explored the role of socioeconomic factors via the human development index (HDI). Based on this framework, we consider potential solutions in equitizing access.

## Methods

This cross-sectional study did not involve human subjects and was conducted using publicly available data sources; therefore, IRB approval was not required.

### Definitions

Subnational Region: The largest defined geographic designation in a country below the national level (analogous to a state or province).

Catchment Area: The geographic region a cardiac surgeon may receive a referral from, defined in this study as the subnational regions for which a cardiac surgeon is closest, irrespective of national borders.

Choropleth Map: A map displaying the distribution of RHD incidence or RHD surgical need using a specified shading pattern.

Incident Case to Capacity Ratio (ICCR): An annualized, calculated ratio of RHD incidence to cardiac surgical case capacity. Ratios greater than one suggest that a region does not have the capacity to meet surgical demand.

RHD Surgical Need: The distance of a subnational region from the nearest cardiac surgeon, calculated by geospatial nearest neighbor analysis, multiplied by the ICCR of the respective catchment area. This computation utilizes distance to represent the challenge patients face, both traveling from distant regions and obtaining definitive screening/referral for RHD.

RHD Surgical Need_HDI_: The surgical need due to RHD of a region standardized by its HDI to account for socioeconomic disparities across the continent.

### Data Sources

Using the Institute for Health Metrics and Evaluation (IHME) 2019 global burden of disease (GBD) tool, RHD incidence data was collected from the Global Health Data Exchange (11). RHD was defined based upon clinical diagnosis and echocardiographic confirmation (12). The full IHME methodology of defining RHD and calculating disease incidence has been previously described (12,13).

As reported previously, The Cardiothoracic Surgery Network (CTSNet) was queried (7) to determine the number of cardiac surgeons and their geographic region of practice for each country in Africa. For greater fidelity, a personal communication with the president of the Pan African Society of Cardiothoracic Surgery yielded the most accurate list of known centers in Africa with cardiac surgery capabilities. This list was cross-referenced with CTSNet data.

Countries were divided into subnational regions. Shapefiles for these regions were collected by country from the Global Administrative Areas Database (GADM) (14) and merged to form a subnational map of Africa in Quantum Geographic Information System (QGIS) 3.2 Lima.

Adult and pediatric (under age 20) populations for these subnational regions were gathered using each country’s census data. If census data for a country was not available or the country did not break down their population by region, population data was collected from the Humanitarian Data Exchange (15), an open-source database maintained by the United Nations Office for the Coordination of Human Affairs (exceptions: subnational populations were unavailable for Equatorial Guinea and the Republic of the Congo, therefore, nationwide metrics were used) (16).

HDI is a metric pioneered by the United Nations Development Programme (UNDP) to utilize a nation’s average life span, educational attainment level, and gross national income per capita to holistically compare nations’ relative level of development (17). The HDI of each country in the analysis was acquired by querying the UNDP’s data center.

Data on geopolitical conflicts in Africa were gathered to contextualize our results using the Council on Foreign Relations global conflict tracker (18).

### Outcomes of Interest

Outcomes were stratified into three geographic levels: continental (composed of all subnational regions), catchment area, and subnational. At the continental level, outcomes included population, number of cardiac surgeons with the corresponding number of catchment areas, RHD incidence, the average distance to a cardiac surgeon, and ICCR. At the level of catchment areas, outcomes included the population served, the average distance to cardiac surgeon(s), and the ICCR. At the subnational level, outcomes included the distribution of RHD cases and their surgical need as well as HDI and surgical need standardized by HDI.

### Analysis

Population, incidence of RHD, and cardiac surgeons were merged into QGIS according to their respective subnational region. Subnational RHD incident cases were calculated for rheumatic heart disease (Equation 1). A choropleth map was then created depicting incident cases of RHD for each subnational region across Africa, stratified by decile.


1 \[
{\mathrm{Incident\;Case}}{{\mathrm{s}}_{{\mathrm{RHD}}}}\; = \;{\mathrm{Subnational\;Population}}\; \times \;\frac{{{\mathrm{Incidenc}}{{\mathrm{e}}_{{\mathrm{RHD}}}}}}{{100,000}}
\]

A nearest neighbor analysis was conducted to determine the nearest cardiac surgeon(s) and corresponding distance for each subnational region in order to construct catchment areas as in prior US-based studies (10,19). The population and the average distance a patient would need to travel were calculated for each catchment area as described previously (9,10).

ICCRs were calculated for each catchment area (Equation 2). The annual average case volumes of cardiac surgeons were derived from STS/AATS taskforce surveys and extrapolated for use in Africa (20).


2 \[
{\mathrm{Incident}}\;{\mathrm{Case}}\;{\mathrm{to}}\;{\mathrm{Capacity}}\;{\mathrm{Rati}}{{\mathrm{o}}_{{\mathrm{Catchment\;Area\;}}}}\; = \;\frac{{\mathop \sum \nolimits_{n = i}^j {\mathrm{Subnational\;Populatio}}{{\mathrm{n}}_i}\; \times \;\frac{{{\mathrm{Incidenc}}{{\mathrm{e}}_{RH{D_i}}}}}{{100,000}}}}{{{\mathrm{\;}}\# {\mathrm{Cardiac\;Surgeons}}\;{\mathrm{\;}}({\mathrm{Catchment\;Area}})\; \times \;{\mathrm{Avg}}.{\mathrm{Annual\;Case\;Vol}}.}}
\]

RHD surgical need for each subnational was calculated (Equation 3). A second choropleth map was generated displaying subnational RHD surgical need, stratified by decile, with lines linking each subnational region to the subnational region of the nearest cardiac surgeon. Subnational regions linked to the same surgeon(s) were within the same catchment area.


3 \[
{\mathrm{Surgical\;Nee}}{{\mathrm{d}}_{{\mathrm{RHD}} - {\mathrm{Dist}}}}\; = \;\frac{{\left( {\mathop \sum \nolimits_{n = i}^j {\mathrm{Subnational\;Populatio}}{{\mathrm{n}}_i}\; \times \;\frac{{{\mathrm{Incidenc}}{{\mathrm{e}}_{RH{D_i}}}}}{{100,000}}} \right)\; \times \;\frac{{{\mathrm{Distance\;to\;Catchment\;Area\;\;Surgeon\;}}\left( {{\mathrm{km}}} \right)}}{{100}}}}{{{\mathrm{\;}}\# {\mathrm{Cardiac\;Surgeons\;}}\;({\mathrm{Catchment\;Area}})\; \times \;{\mathrm{Avg}}.{\mathrm{Annual\;Case\;Vol}}.}}
\]

A map stratifying HDI by decile across Africa was created for visualization of socioeconomic disparities. RHD surgical need of each region was then standardized by its respective HDI (Equation 4), and a choropleth map was constructed to investigate how incorporating socioeconomic variability affected the previous RHD surgical need model.


4 \[
{\mathrm{RHD\;Surgical\;Nee}}{{\mathrm{d}}_{{\mathrm{HDI}}}}\; = \;{\mathrm{Surgical\;Nee}}{{\mathrm{d}}_{{\mathrm{RHD}} - {\mathrm{Dist}}}}\; \times \;\left( {1 - {\mathrm{HDI}}} \right)
\]

## Results

### Continent

The African continent was estimated to have a population of 1,298,333,309 people—658,283,537 adults (50.7%) and 640,049,772 (49.3%) children under the age of 20. There were 509 cardiac surgeons practicing in 74 subnational regions (corresponding to 74 catchment areas). There were 1,027,974 incident cases of RHD annually. The average distance to the nearest cardiac surgeon, weighted by population, was 312.01 km (193.87 miles). The ICCR due to RHD for the African continent was 10.64.

### Catchment Areas

**Figure 1:**
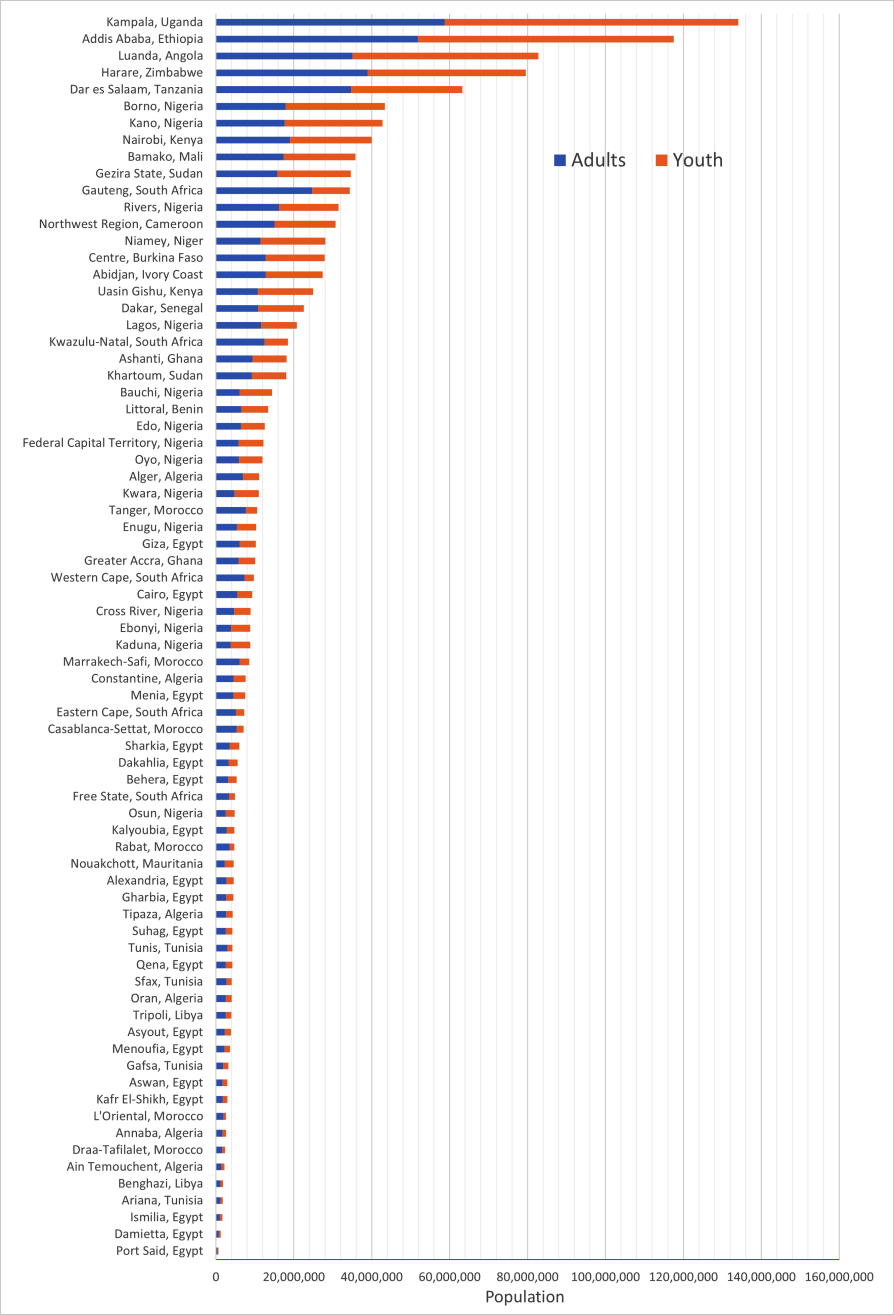
Populations served by the 74 cardiac surgical catchment areas across Africa.

The population of each catchment area ranged from 628,440 individuals (served by one cardiac surgeon in Port Said, Egypt) to 134,074,264 individuals (served by three cardiac surgeons in Kampala, Uganda). The populations of each catchment area stratified into youth and adult age groups are shown in Figure 1.

**Figure 2:**
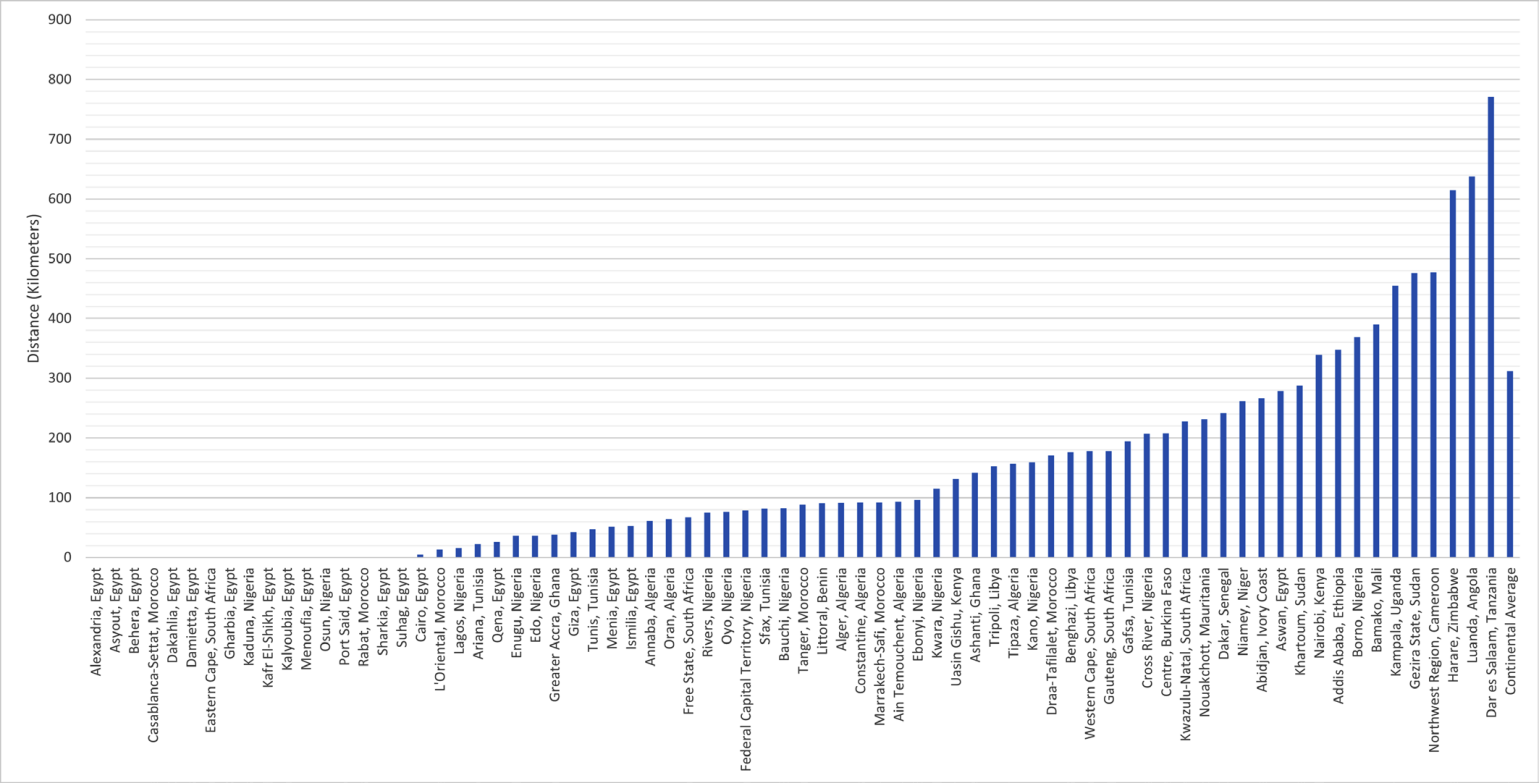
Average distance a patient in each catchment area would travel to see a cardiac surgeon, weighted by population.

The average distance a patient would need to travel (Figure 2) to see a cardiac surgeon in their catchment area, weighted by population, ranged from one kilometer (0.62 miles) to 818.17 kilometers (508.39 miles).

**Figure 3:**
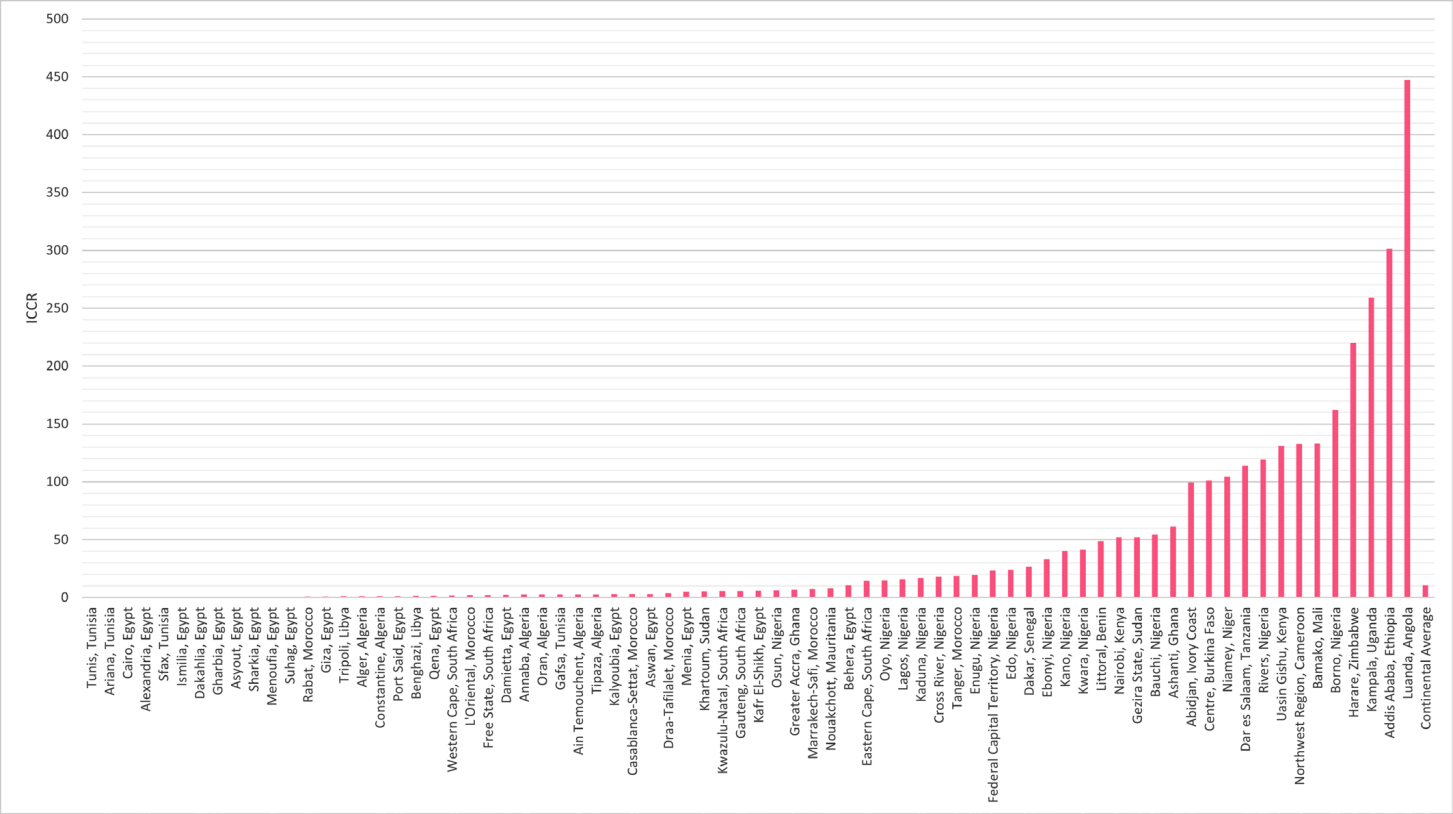
ICCR for each of the 74 cardiac surgical catchment areas across Africa.

The ICCR for each catchment area ranged from 0.04 for regions served by Tunis, Tunisia, to 447.30 for regions served by Luanda, Angola (Figure 3).

### Subnational

**Figure 4:**
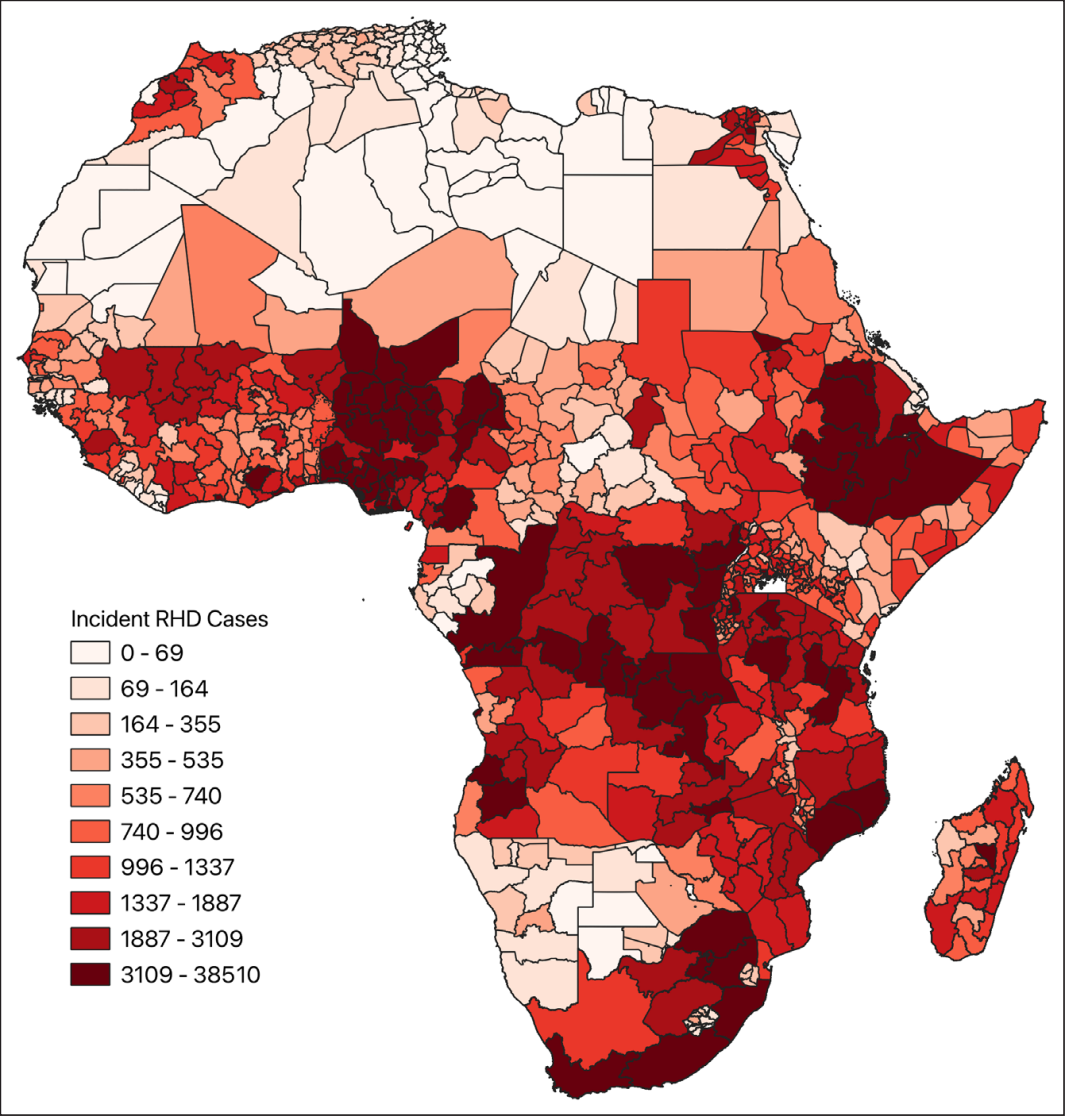
Choropleth map depicting incident cases of RHD across the subnational regions of Africa.

A total of 779 subnational regions from 54 countries were included in the analysis. The estimated median was 739.34 (IQR: 227.54–1583.25) incident cases, and the mean was 1,317.92 ± 81.05 incident cases of RHD across all subnational regions. The estimated annual number of index RHD cases by subnational region ranged from 3.16 cases (min–max: 2.72–3.62) in Tozeur, Tunisia, to 38,509.99 (min–max: 28,205.24–50,363.95) in Oromia, Ethiopia. A choropleth map depicting the incidence of RHD in each subnational region demonstrates the highest case density occurring in West Africa, the Horn of Africa, Southern Africa, and pockets of Central Sub-Saharan Africa (Figure 4).

**Figure 5:**
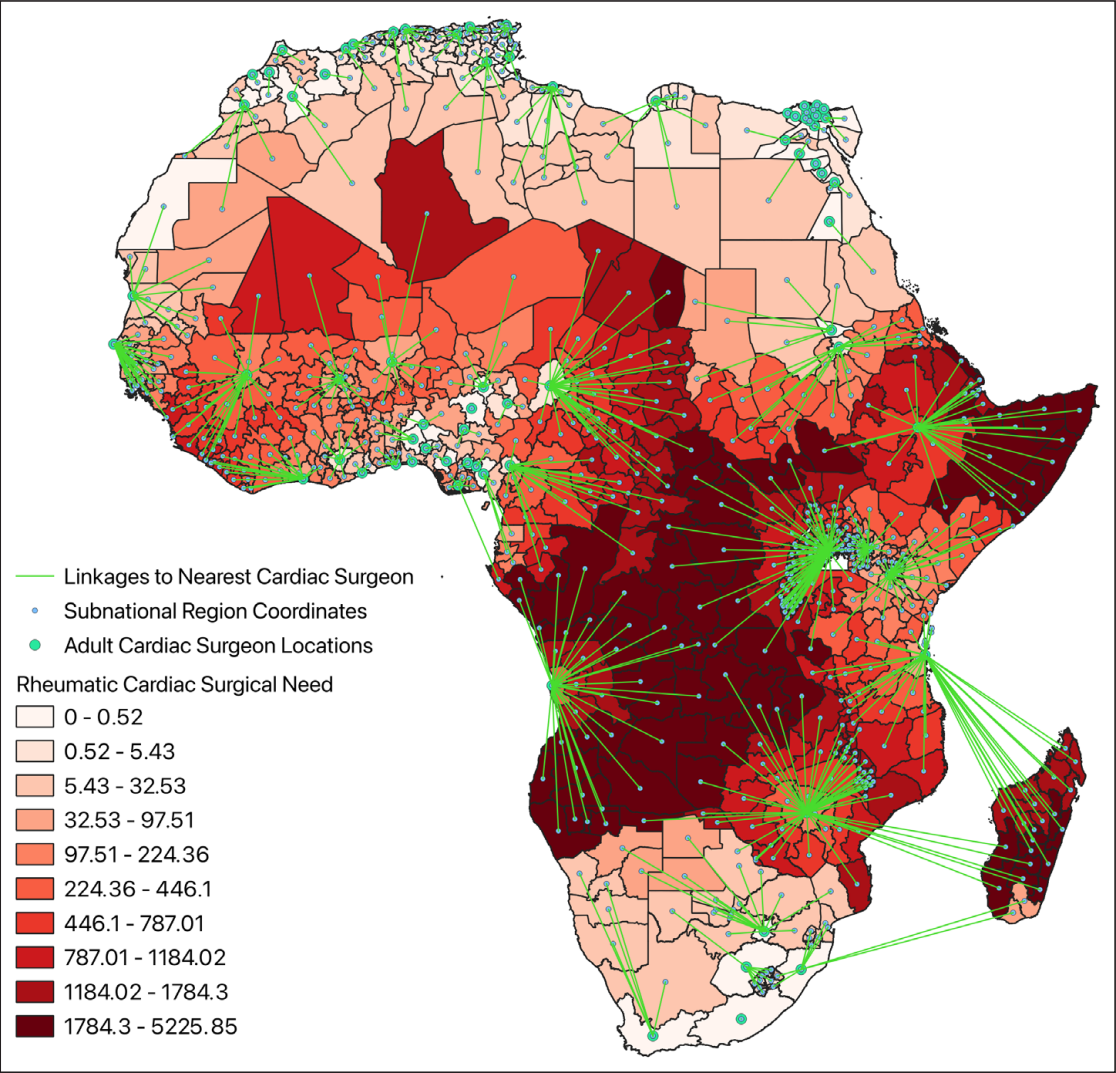
Choropleth map of cardiac surgical need due to RHD across the subnational regions of Africa with linkages to the nearest cardiac surgeon.

RHD surgical need for each subnational region, incorporating distance from the nearest cardiac surgeon, is shown in Figure 5, where green lines link each subnational region to the location of the nearest cardiac surgeon. Subnational regions linked to the same cardiac surgeon location comprise the same catchment area. Areas of greatest RHD surgical need occurred in the Congo Basin, the coast of the Horn of Africa, and Madagascar. RHD surgical need was often greatest in areas most distant from surgeons.

**Figure 6:**
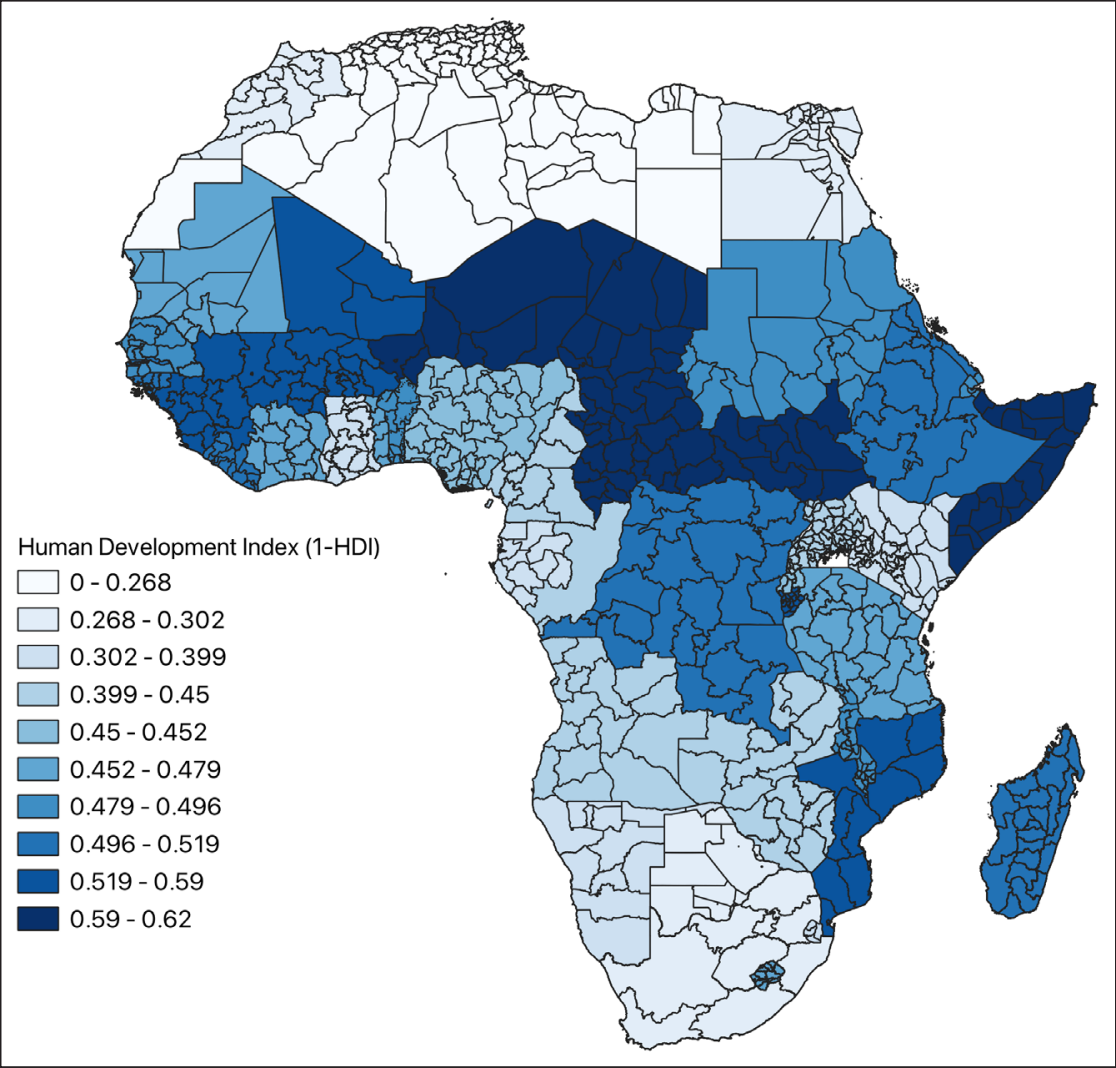
Map of distribution of HDI of the subnational regions across Africa.

**Figure 7:**
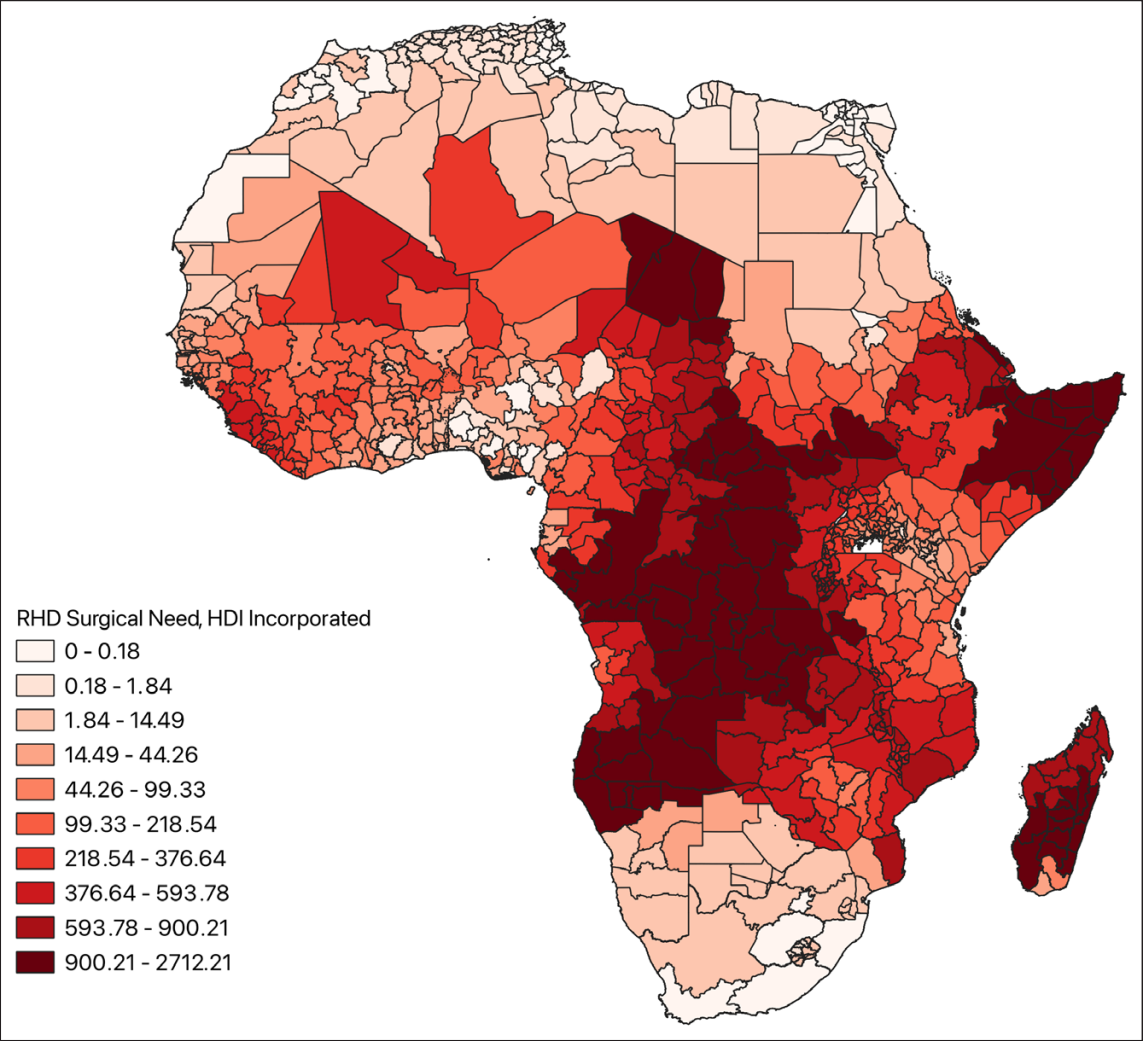
Choropleth map of RHD surgical need across Africa incorporating regional HDI.

HDI across all regions included in the analysis is shown in Figure 6. Regions with the greatest development occurred in North Africa above the Sahara Desert and in South Africa, whereas areas with the least development clustered in central Sub-Saharan Africa and the Horn of Africa. RHD surgical need standardized by HDI is depicted in Figure 7. Areas of greatest surgical need incorporating HDI similarly occurred in the Congo Basin, the coast of the Horn of Africa, and Madagascar.

## Discussion

To our knowledge, we are the first to report that across 779 subnational regions comprising 74 catchment areas across Africa, <10% of RHD cases can be accommodated by current surgical resources. We are also the first to estimate that the average distance travelled for cardiac surgical care is 312.01 km (193.87 miles), and the highest RHD surgical need is not isolated to areas with the greatest incidence of RHD. With these results in mind, we propose concepts to consider in solution management. Prior work assessing cardiac surgical need in Africa has normalized the number of cardiac surgeons or the number of centers to national population to estimate access. While an important first step, these studies serve only to confirm the intuition that cardiac surgical care in Africa is limited. These works do not fully capture the extent to which surgeons and hospitals are overwhelmed by potential cases nor account for the geographical scale of Africa. These are necessary considerations given Price et al. showing that the cost of transportation alone is a major barrier to seeking care (21) and the African Development Bank Group’s findings that over 50% of roads in Africa are unpaved (22).

Unlike other forms of cardiovascular disease, RHD is preventable through the use of antibiotics for primary and secondary prophylaxis (1). Despite the recognition of the devastating effects of RHD by public health authorities, including the World Health Organization, programs promoting the screening and prevention of RHD are not widespread. Even where programs exist, adherence to prophylactic regimens is suboptimal (23). Promotion of programs to screen for/treat RHD (24,25) and enhance antibiotic adherence (23) may be an impactful way to reduce the burden of RHD on developing health systems. This is especially pertinent in regions where access to cardiac surgical intervention for severe cases remains grossly limited, as we have shown.

The paucity of funding cardiac surgery receives from the largest donor of global health initiatives in the world (the US government) is noteworthy (26). In 2016, 0.26% of the federal US budget (~$10.3 billion) was earmarked for global health initiatives (27). Of these funds, those directed to noncommunicable diseases, including cardiovascular diseases and surgical conditions, received only 3% of earmarked allocations (~$3M) (28,29,30). In comparison, HIV, tuberculosis, and malaria received over 50% of these funds (29). This is despite the mortality associated with cardiovascular and surgical conditions in Africa accounting for five times as many deaths as HIV, tuberculosis, and malaria combined (31,32,33). While this is due to the groundbreaking advancements in the management of infectious diseases and efforts of global health champions over the past four decades, perhaps it is time to restructure funding models to better accommodate cardiovascular and surgical diseases.

In the absence of widespread, independent, and sufficient cardiac surgical services in Africa, there has been emphasis on utilizing non-governmental organizations (NGOs) to assuage the dire need gap for cardiac surgical services that exists in LMICs. Previous studies have identified approximately 86 NGOs providing cardiac surgery in 96 LMICs (34,35), performing an average of 10,212 cases annually across the globe, with the median performed by individual NGOs being 79 cases (34). While this is a noble endeavor, improving the lives of recipients, from a health systems perspective, it is an inefficient way to bridge the need gap given our estimate of ~930,000 incident cases of RHD that surpass surgical case capacity in Africa alone. Moreover, in addition to their limited impact, NGOs face unique challenges limiting their efficacy, including mistrust between transient NGOs and local staff, post-operative follow-up, management of complications when surgical staff reside overseas, and disparate medical record keeping (36,37).

One of the most promising, longitudinal methods of augmenting access to cardiac surgical services while avoiding the pitfalls of NGOs has been the establishment of *twinning programs*. Within these programs, cardiac surgical centers of excellence in resource-rich settings are paired with developing cardiac programs in resource-limited settings (38). This longitudinal partnership (typically >5 years) fosters bidirectional learning and skill development, which can include formal instruction (i.e., fellowship, perfusionist training, etc.), material support aimed at sustainability, and preparing developing centers to become training centers themselves (38). However, it is not enough to simply develop these centers; our results and similar studies in the U.S. suggest geographically informed cardiac surgical centers are imperative to favorably distribute care and achieve better outcomes (9,10,19).

Providing cardiac care to African patients in remote locations will require innovation while hospitals with these capabilities are established. Previous innovations such as Mercy Ships have helped address the global surgical crisis for citizens of coastal LMICs (39), and general as well as surgical subspecialties have overcome similar land-based challenges with teams of local surgeons leading mobile surgical units (40,41). Adoption of these ideas in the cardiac context could be used to augment ongoing screening missions to remote locations performed by NGOs (42) and provide routine treatment as well as follow-up care if performed in a serial, systematic manner. Ultimately, while intended as a temporizing measure, this would also provide graduates from nascent African cardiac programs a flexible practice in addition to expanding access to care (40).

Establishing equitable cardiac surgical care across Africa is vital, and we propose initial efforts may be most beneficial through supporting: 1) existing secondary antibiotic prophylactic programs to decrease the incidence of RHD directly, 2) restructuring of global health funding allocations, 3) a shift away from reliance on NGO-provided cardiac surgical services towards capacity building of all facets of the cardiac surgical workforce in geographically primed locations, and 4) innovative delivery of treatment to remote patients.

### Limitations

Our analysis has several limitations. We fully acknowledge our methodology of collecting surgeon data from CTSNet is imperfect. However, this methodology has been used previously in other large-scale geographic analyses of access to cardiac surgical services (7) and is necessary as no other continent-wide registries of cardiothoracic surgeons exist. It also avoids the availability bias inherent in relying on information from regional contacts.

Catchment areas were constructed irrespective of national borders; assuming patients would be able to cross borders without difficulty due to medical need may not reflect geopolitical reality. With this understanding, Supplemental Table 1 characterizes locations and geopolitical conflicts that may especially limit the mobility of patients. These conflicts occurred primarily in Central Sub-Saharan regions of the lowest development and the greatest RHD surgical need, likely limiting access to cardiac surgical intervention in these regions even further. While this limits the analysis, it points to a potential solution. Large swaths of the populations in these areas have been displaced due to violence and live in refugee camps with foreign aid. Screening and secondary prophylaxis regimens would more easily be implemented in these areas, and the sickest refugees with RHD could potentially be fast-tracked for asylum due to medical necessity.

Additionally, we calculated distances in a linear point-to-point fashion, not accounting for actual road/transit networks patients might use, which are likely further and inefficient. However, given that greater than 50% of roads are unpaved and the cost of air travel is prohibitive for a majority of citizens (22), this method is a reasonable approximation. Moreover, we assumed each surgeon could meet the average U.S. case volume and their case volume was composed solely of RHD, likely resulting in an underestimation of RHD surgical need. Finally, we assumed that the national estimates of RHD incidence were homogenous throughout a country.

HDI was also included in the analysis in order to explore the impacts of socioeconomic disparities on RHD surgical need. When comparing Figure 5 and Figure 7, it appears HDI had minimal impact on the relative RHD surgical need across regions included in the analysis. This is likely because lesser developed areas have a greater incidence of RHD and are more isolated from cardiac surgeons due to underdeveloped health infrastructure, which is likely correlated with reduced lifespans of respective populaces. Moreover, the dearth of cardiac surgeons is likely associated with decreased levels of educational attainment. This would imply RHD surgical need incorporates aspects of development in its calculation, with areas of the greatest need concurrently being the least developed. Adding HDI to the analysis only reiterates this fact. Another plausible explanation could be that despite the relative differences in HDI depicted in Figure 6, the absolute differences in HDI are not large enough across regions to have a meaningful impact on the analysis.

### Conclusions

Across Africa, access to cardiac surgical care is limited despite the high incidence of RHD. While nearly all areas will benefit from increasing cardiac surgical capacity, attention should be paid to the geographic distribution of surgeons, as limited access to cardiac surgery is compounded by distance. Support of screening and antibiotic programs, reallocating global health funds, emphasizing the development of geographically informed cardiac centers, and innovative surgical distribution schemes are needed to address these needs.

## Additional File

The additional file for this article can be found as follows:

10.5334/gh.1394.s1 Supplementary Table 1. Geopolitical Conflicts Across Africa Potentially Limiting Civilian Movement.

## Abbreviations

ABTS: American Board of Thoracic Surgery

CTSNet: The Cardiothoracic Surgery Network

GADM: Global Administrative Areas

GBD: Global Burden of Disease

HDI: Human Development Index

ICCR: Incident Case to Capacity Ratio

IHME: Institute for Health Metrics and Evaluation

LMIC: Low- to Middle-Income Country

NGO: Non-Governmental Organization

QGIS: Quantum Geographic Information System

RHD: Rheumatic Heart Disease

UNDP: United Nations Development Programme
