# Supplementary material for: Cardiac Surgery to Manage Rheumatic Heart Disease in Africa Is Complex — a Geographic Perspective
Source: Glob Heart. 2025 Feb 3;20(1):10. doi: 10.5334/gh.1394 (PMC11804176; doi:10.5334/gh.1394)
Supplement: Supplementary Table 1. — Geopolitical Conflicts Across Africa Potentially Limiting Civilian Movement. [file gh-20-1-1394-s1.pdf]

Supplementary Table 1. Geopolitical Conflicts Across Africa Potentially Limiting Civilian Movement

**Region**	**Conflict Description**
Libya	Civil war with ceasefire – the nation is now in civil unrest as democratic election of governmental leadership has been indefinitely postponed.
The Sahel	Violent extremism propagated by organizations including Jama'at Nusrat al-Islam wal Muslimeen (JNIM), Islamic State in the Greater Sahara (ISGS), Islamic State in the West African Province (ISWAP) against local governments and civilians primarily in Mali, Niger, Chad, and Burkina Faso limiting transience of citizens.
Central African Republic	Ongoing civil war with rebel groups opposing current regime and millions of civilians displaced by conflict and United Nations aid workers facing violence.
Sudan	Civil war with over 8 million civilians displaced and reports of human rights violations. Despite numerous attempts at negotiation ceasefires have occurred.
South Sudan	After a period of civil war and formation of unity government, the new regime faces instability and violence from opposition groups. Due to displacement during the civil war two-thirds of the population live in refugee camps facing violence from opposition groups and food insecurity.
Ethiopia	Civil unrest as the federal government battles opposition of one nation's provinces and ethnic violence. Additionally, war with Eritrea add to tense regional relations with neighboring nations.
Somalia	Al-Shabaab acts as Al-Qaeda's strongest affiliates exploiting the Somalian government's limited state capacity to launch attacks against government forces, international peacekeepers, and civilians to overthrow the federal government. Due to Al-Shabaab's terrorist efforts to attack neighboring nation's immigration of Somalian civilians is limited.
The Democratic Republic of the Congo	Political instability with violent clashed between the ruling military and insurgents in the Eastern region of the country. Additionally, tense relations with Uganda and Rwanda limit movement across borders.
